# Novel Device to Sample the Esophageal Microbiome—The Esophageal String Test

**DOI:** 10.1371/journal.pone.0042938

**Published:** 2012-09-05

**Authors:** Sophie A. Fillon, J. Kirk Harris, Brandie D. Wagner, Caleb J. Kelly, Mark J. Stevens, Wendy Moore, Rui Fang, Shauna Schroeder, Joanne C. Masterson, Charles E. Robertson, Norman R. Pace, Steven J. Ackerman, Glenn T. Furuta

**Affiliations:** 1 Department of Pediatrics, Section of Gastroenterology, Hepatology and Nutrition, Digestive Health Institute, Children's Hospital Colorado, Gastrointestinal Eosinophilic Diseases Program, Mucosal Inflammation Program, University of Colorado Denver, School of Medicine, Aurora, Colorado, United States of America; 2 Department of Pediatrics, Division of Pulmonology, University of Colorado Denver, School of Medicine, Aurora, Colorado, United States of America; 3 Department of Biostatistics and Informatics, Colorado School of Public Health, University of Colorado, Aurora, Colorado, United States of America; 4 Department of Molecular Cellular and Developmental Biology, University of Colorado Boulder, Colorado, United States of America; 5 Department of Biochemistry and Molecular Genetics, University of Illinois at Chicago, Chicago, Illinois, United States of America; University of Colorado Denver, United States of America

## Abstract

A growing number of studies implicate the microbiome in the pathogenesis of intestinal inflammation. Previous work has shown that adults with esophagitis related to gastroesophageal reflux disease have altered esophageal microbiota compared to those who do not have esophagitis. In these studies, sampling of the esophageal microbiome was accomplished by isolating DNA from esophageal biopsies obtained at the time of upper endoscopy. The aim of the current study was to identify the esophageal microbiome in pediatric individuals with normal esophageal mucosa using a minimally invasive, capsule-based string technology, the Enterotest™. We used the proximal segment of the Enterotest string to sample the esophagus, and term this the “Esophageal String Test” (EST). We hypothesized that the less invasive EST would capture mucosal adherent bacteria present in the esophagus in a similar fashion as mucosal biopsy. EST samples and mucosal biopsies were collected from children with no esophageal inflammation (n = 15) and their microbiome composition determined by 16S rRNA gene sequencing. Microbiota from esophageal biopsies and ESTs produced nearly identical profiles of bacterial genera and were different from the bacterial contents of samples collected from the nasal and oral cavity. We conclude that the minimally invasive EST can serve as a useful device for study of the esophageal microbiome.

## Introduction

Recent studies of the human microbiome have provided robust support for the important role of the microbiome in health and disease [1], [2]. Whereas a significant body of work defines the intestinal microbiology, few studies have investigated the esophageal microbiome. To date, these studies utilized mucosal biopsy samples that were obtained at the time of endoscopic procedures [3], [4], [5], [6], [7], [8]. While technically feasible, this methodology is invasive, time consuming, costly, and carries potential complications. In addition, repeated and frequent sampling following therapeutic interventions are impractical and the full characterization of esophageal mucosal microbiome is limited to a ∼3 mm section of procured tissue.

To address this issue, we utilized the proximal section of a minimally invasive device, the Enterotest™, to sample the esophageal mucosal microbial microenvironment. This capsule-based string technology involves the subject swallowing a weighted gelatin capsule filled with a string in which the proximal end is taped to the cheek and the trailing end, with the capsule attached, travels to the duodenum. At the time of endoscopy, 12–18 hours after string placement, the tape is removed from the cheek and the string is retrieved. The aim of this study was to harvest adherent secretions from the proximal portion of the string that was situated in the esophagus and analyze ribosomal RNA gene sequences. We hypothesized that analogous to fecal sampling, the intraluminal contents in the esophagus would be reflective of the mucosal microbial environment present on esophageal mucosal tissue.

## Materials and Methods

### Esophageal string test (EST)

This study was approved by the Colorado Multiple Institutional Review Board (COMIRB). Written informed consent and HIPPA authorization were obtained from all participants or from parents or legal guardians of participants younger than 18 years. Assent was obtained from all participants under 18 years.

Children 7–20 years of age who were undergoing an endoscopy with biopsy to determine causes of abdominal pain, vomiting, growth failure, or dysphagia were enrolled in this study. Exclusion criteria included a history of esophageal stricture, narrowing, fundoplication, gelatin allergy or other co-morbidities with increase risk (bleeding diatheses, connective tissue diseases) of endoscopic complications. Review of endoscopic and pathology records were performed to determine normal diagnoses. All children with normal mucosal biopsies were included in this study report.

The night prior to the endoscopy, subjects swallowed an Enterotest™ capsule (Figure S1). This technology was developed and began successful use in the 1970's to identify small intestinal infections (e.g. *Giardia lamblia*) [9], [10], [11] and was subsequently used to sample gastric contents for *Helicobacter pylori* [12], [13]. The pediatric Enterotest™ consists of a weighted gelatin capsule filled with 90 cm of nylon string. The proximal end of the string (∼10 cm) extrudes from one end of the capsule, which is held and taped to the cheek after the capsule is swallowed. When swallowed, the capsule deploys the capture material, the nylon string, during its passage through the esophageal, gastric and duodenal lumen and is finally released into the duodenum where it eventually is passed in the stool. No food or medications were consumed after the EST was in place. The EST was removed before or just after induction of general anesthesia. Locations of esophageal and gastric segments of the EST were determined using a combination of pH indicator sticks supplied with the tests, and by measuring the distance to the lower esophageal sphincter at the time of endoscopy.

### Microbiome identification

Mucosal biopsies were then obtained from the middle to distal part of the esophagus. Nasal swabs, a 2 cm segment of oral string and a 2 cm segment of the middle part of the esophageal string were collected from each subject. Biopsies, swabs and string samples were snap frozen in liquid nitrogen and kept at −80°C until DNA extraction.

DNA from all samples was extracted using Qiagen DNAeasy Extraction Kits for blood and tissue according to manufacturer's specifications (Qiagen, Valencia, CA). DNA was amplified in triplicate with barcoded PCR primers that include adaptors for the Roche 454 sequencing platform [14]. Negative PCR controls were performed for each barcode, and PCR was repeated for any sample where the negative control was positive. Amplicons were pooled after normalization of DNA concentration [15], and sequenced using the Roche 454 FLX platform according to manufacturer's specifications (Roche, Branford, CT). Sequence data were assigned to samples of origin using bar code sequences added during PCR, and screened for basic quality defects (short sequences <200 nucleotides in length, >1 sequence ambiguity, best read with quality ≥20 over a 10-nucleotide moving window) by the program BARTAB [16]. Non-bacterial sequences were removed from datasets by requiring a close match with a bacterial rRNA secondary structure model within Infernal [17]. Sequences identified as potential chimeras by ChimeraSlayer [18] were also removed from datasets. The Ribosomal Database Project Classifier software was used to make taxonomic assignments [19]. Taxonomic information was used to construct sequence groups with identical taxonomic rank, which were used for bacterial community analyses, and to identify specific bacteria that were differentially present between locations.

### Statistical analyses

Microbiome DNA results obtained from mucosal biopsy samples were considered the “gold standard” reference; microbiome results obtained from the EST samples were compared to those obtained from the biopsies in all statistical analyses. We defined common genera as ≥1% relative abundance in any sample and rare genera as <1% relative abundance in any sample.

Descriptive statistics included common ecological parameters such as Shannon diversity [20]. For individual taxa, the percentage of samples where the taxa was present, and the median value of the relative abundance, are presented. Comparisons between two groups were performed individually for each taxa using the paired version of the two-part test and the negative log p-values are displayed using the Manhattan plot [21]. The two-part test is the sum of two squared tests statistics, one comparing the proportion of non-zero counts and one comparing the medians of the non-zero counts. All analyses were performed using SAS Version 9.2 software (SAS Institute Inc.: Cary, NC, 2008).

## Results

### Sample collection

Fifteen subjects (female, n = 10) were enrolled in this study ranging in age from 11 to 18 (median age of 15) with the following ethnic diversity; twelve White, one Black, one Hispanic and one other. Five subjects were on proton pump inhibitor (PPI) for heartburn at the time of the study, two were on inhaled steroids for asthma or rhinitis, three were on food elimination diet, and one was on inhaled steroid in addition to PPI and food elimination diet. All subjects had normal histological biopsy findings. Thirteen nasal swabs and fifteen oral strings, ESTs and biopsies were collected. Bacterial ribosomal RNA gene amplification products from mucosal biopsies and from the nasal cavity, oral cavity and EST were visualized by gel electrophoresis (Figure S2).

### Microbiome complexity is similar in oral, nasal and esophageal microenvironments

**Figure 1:**
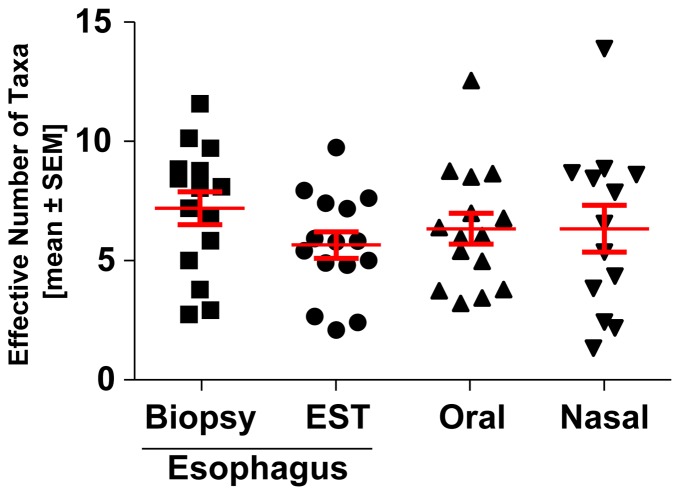
Microbiome phylum-level diversity is similar in esophageal, oral and nasal microenvironments. Microbiome diversity was measured from DNA samples taken from the esophageal mucosa, oral and nasal samples as identified using 454 pyrosequencing. Each point represents the number of Taxa present in each sample. The mean ± standard error of the mean are presented.

To determine the extent of the bacterial diversity of the different microenvironments, we evaluated the number of phylum-level taxa present in the oral, nasal and esophageal samples. No significant differences in the complexity of microbiome diversity were observed between the nasal swab, oral string or esophageal biopsy or EST samples at this broad taxonomic level (Figure 1). Variation in this pattern emerged at subphylum levels. In addition, similar numbers of bacterial phyla occur in each location.

### EST and mucosal biopsy capture similar bacterial assemblages

**Figure 2:**
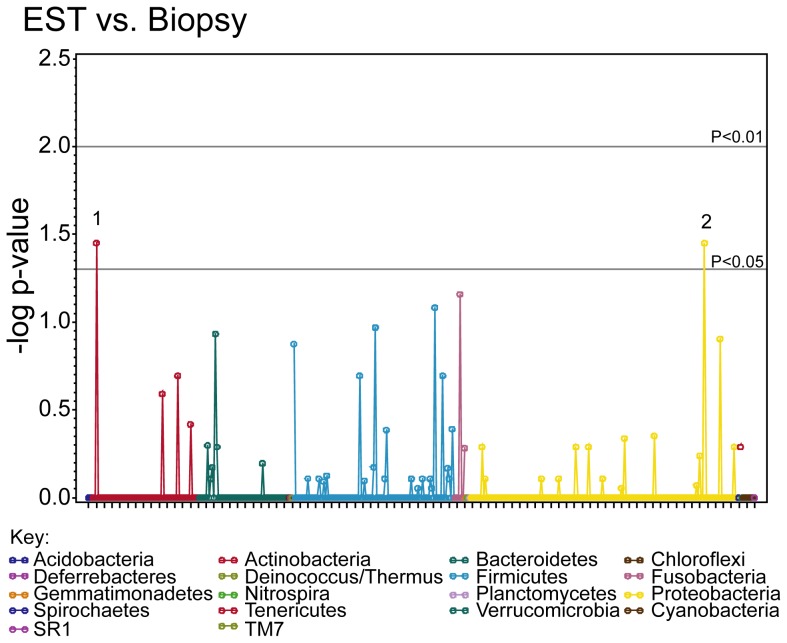
Esophageal genera captured on mucosal biopsies and ESTs are similar. Bacterial taxa were compared from 15 matched biopsy and EST samples, the negative log p-value from this comparison is displayed for each taxa. The gray horizontal lines indicate significant differences (p<0.05 and p<0.01). The phyla are represented in different colors as in the key. Genera that were significantly different are numbered. Numbers represent: 1, *Actinomyces*; 2, *Pasteurella*. *2*, had higher relative abundance in EST versus biopsy.

To determine the ability of the EST to capture the esophageal microbiome, we procured DNA samples from paired esophageal mucosal biopsies and ESTs and then compared the sequence patterns in each. As shown in Manhattan plots (Figure 2), only 2 statistically significant differences were identified in comparing biopsy and EST, *Pasteurella* and *Actinomyces*. *Pasteurella* was increased in EST samples compared to mucosal biopsies (P = 0.04), whereas *Actinomyces* was detected more often in biopsy samples, although only in small quantities (relative abundance less than 5%, P = 0.04). These results support the use of the EST to sample the microbiome as compared to the “gold standard”, the mucosal biopsy.

### Phylum-level contents of esophageal biopsy and EST microbiomes are similar

**Figure 3:**
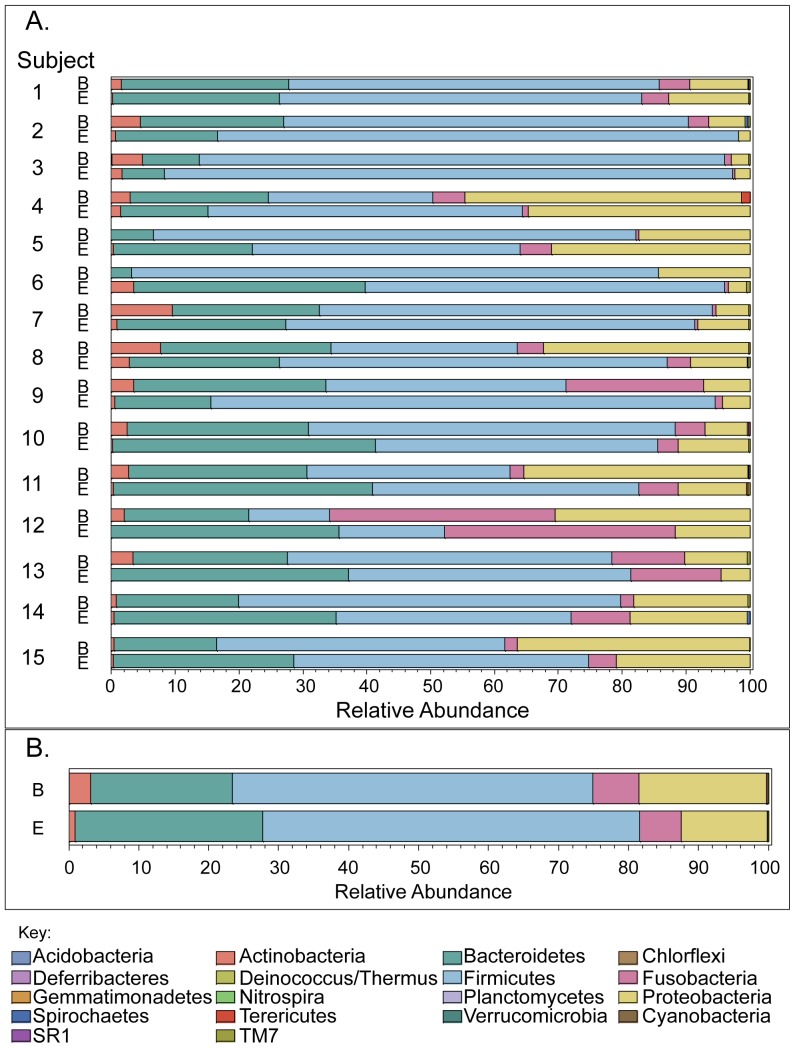
Esophageal phyla captured on mucosal biopsies and ESTs are similar. A. Bar graphs indicate phylum detected in 15 matched biopsy (B) and EST samples (E) with each line corresponding to one subject sample. Each phylum is indicated in a different color. The width of the bar corresponds to the relative phylum abundance. B. Bar graphs present the aggregate of all subjects (1–15) of the relative phylum abundance detected in biopsies (B) and ESTs (E).

We next compared the individual subject esophageal phyla present in the matching mucosal biopsies and ESTs (Figure 3). Individually, the relative abundance was similar for *Actinobacteria*, *Bacteroidetes*, *Firmicutes*, *Fusobacteria* and *Proteobacteria* in both biopsy and EST (Figure 3A). In addition, the aggregate compilation of data from all subjects analyzed also showed virtually identical patterns of relative abundance in these predominant phyla present in biopsies and ESTs (Figure 3B).

### Similar genera in the esophageal biopsy and EST microbiomes

**Figure 4:**
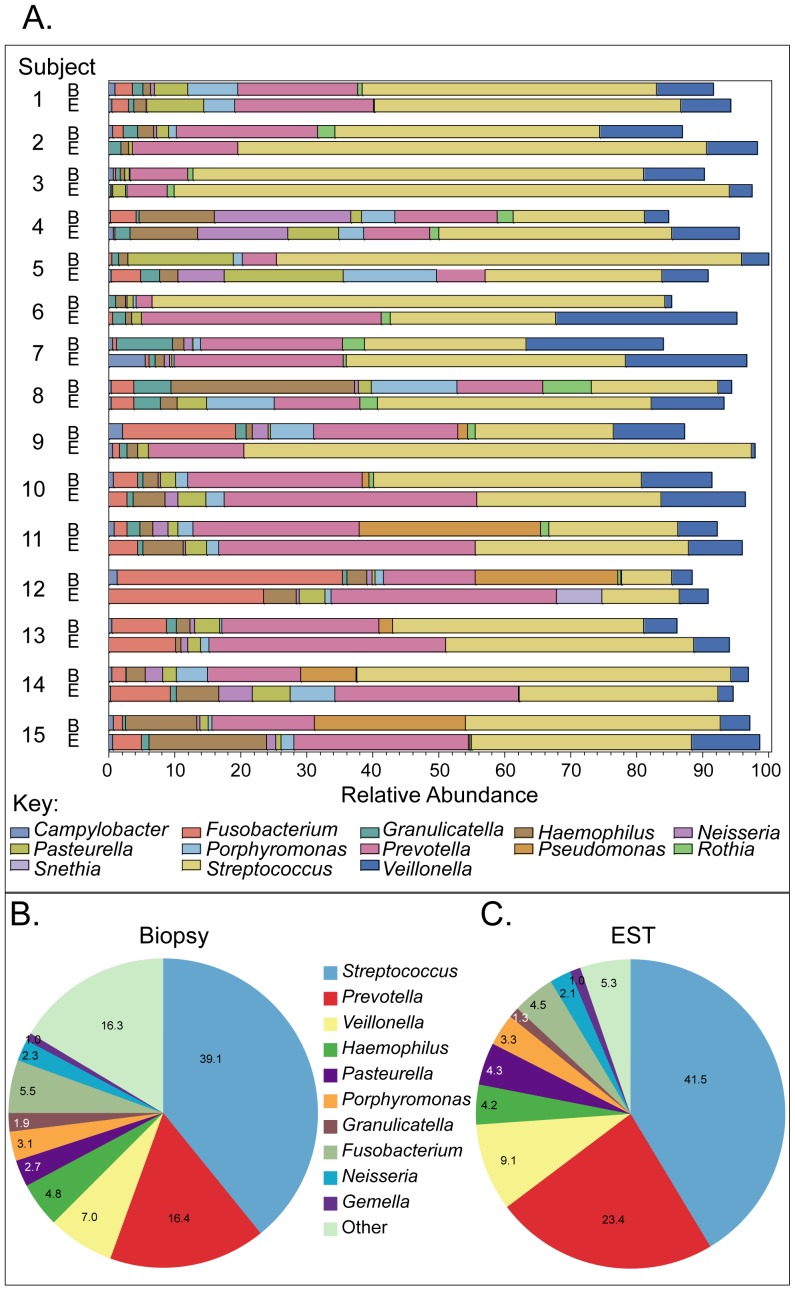
Similar genera in the esophageal biopsy and EST microbiomes. A. Bar graphs indicate genera detected in 15 matched biopsy (B) and EST samples (E) with each line corresponding to one subject sample. Each genus is indicated in a different color. The width of the bar corresponds to the relative genus abundance. B. Pie chart composed of the 10 most prevalent genera in biopsies (n = 15). C. Pie chart composed of the 10 most prevalent genera in ESTs (n = 15). The average percentage of each genus is indicated based on the total bacterial population measured.

To determine whether a similar pattern of microbiome population was present at the genus level, we again compared the individual subjects EST and mucosal biopsy samples (Figure 4A). Thirty-one genera were identified in both EST and biopsy samples, with *Streptococcus*, *Prevotella*, and *Veillonella* being the 3 most common (Figure 4B–C). Comparison of the top 10 genera present on 15 biopsies (Figure 4B) and corresponding ESTs (Figure 4C) indicated that the composition was similar across the two sample sources.

**Figure 5:**
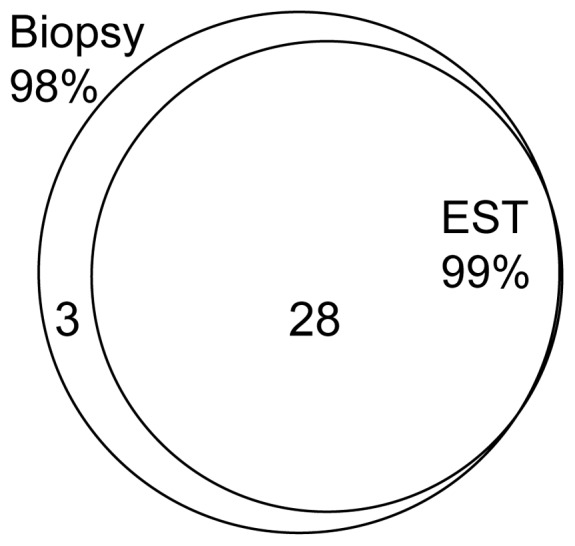
Percentage of common genera captured on mucosal biopsies and ESTs. Venn diagram indicating the overlap between biopsies and ESTs for the common genera identified (≥1% relative abundance).

The overlap between the genera present on the biopsies and the EST is higher than 75% (Figure 5). The number of genera was higher on biopsies than on ESTs (97 vs. 49). The most common genera defined as ≥1% relative abundance in any samples were largely shared between the biopsy and EST (Figure 5), with only three genera that were discordant (3 in biopsy only, Table 1). Table 1 summarizes the common genera identified in this study and the proportion of subjects where each genus was identified along with the median relative abundance of each group. Genera were ordered based on a composite rank of proportion of samples positive and relative abundance independently for the two sample types. Table 1 is ordered by the rank present in the biopsy samples. Rare taxa were different, with a large proportion identified in the biopsy only, 50 compared to 21 in EST (4 in EST only). Rare taxa represented only 2% of genera in biopsy and 1% in EST. These rare genera were detected in low numbers of samples (38/48, 79%, were seen in a single sample).

**Table 1:** Prominent Genera of Esophageal Microbiome.

		Biopsy			EST			
Phyla	Genera	Rank	Median	Percent Detected*	Rank	Median	Percent Detected*	Disagreement (%)†
Firmicutes	*Streptococcus*	1	38.7	100	1	35.3	100	0
Bacteroidetes	*Prevotella*	2	15.5	100	2	25.6	100	0
Firmicutes	*Veillonella*	3	5.1	100	3	7.7	100	0
Proteobacteria	*Haemophilus*	4	2.1	100	5	2.5	100	0
Proteobacteria	***Pasteurella***	5	1.6	100	4	3.3	100	0
Bacteroidetes	*Porphyromonas*	6	1.4	100	9	1.8	80	20
Firmicutes	*Granulicatella*	7	1.0	100	8	1.0	87	13
Fusobacteria	*Fusobacterium*	8	2.1	93	6	2.8	87	30
Proteobacteria	*Neisseria*	9	0.6	93	10	0.5	67	40
Firmicutes	*Gemella*	10	0.6	93	7	0.9	93	13
Actinobacteria	*Rothia*	11	0.7	73	15	0.1	53	47
Fusobacteria	*Leptotrichia*	12	0.7	73	11	0.3	67	33
Proteobacteria	*Campylobacter*	13	0.5	87	13	0.2	60	40
Actinobacteria	***Actinomyces***	14	0.5	80	16	0	47	47
Firmicutes	*Oribacterium*	15	0.3	60	12	0.2	67	33
Firmicutes	*Solobacterium*	16	0.2	60	18	0	33	40
Actinobacteria	*Atopobium*	17	0.2	53	19	0	27	53
Firmicutes	*Megasphaera*	18	0.2	53	21	0	20	33
Firmicutes	*Peptostreptococcus*	19	0.1	53	22	0	20	60
Bacteroidetes	*Capnocytophaga*	20	0	47	17	0	47	27
Proteobacteria	*Pseudomonas*	21	0	47	27	0	7	40
Proteobacteria	*Aggregatibacter*	22	0	40	14	0.1	53	27
Tenericutes	*Mycoplasma*	23	0	27	28	0	7	20
Proteobacteria	*Delftia*	24	0	20	30	0	nd	20
Proteobacteria	*Kingella*	25	0	20	23	0	20	27
Proteobacteria	*Phocoenobacter*	26	0	20	24	0	13	20
Proteobacteria	*Stenotrophomonas*	27	0	20	31	0	nd	20
Fusobacteria	*Streptobacillus*	28	0	13	20	0	27	13
Proteobacteria	*Bibersteinia*	29	0	13	26	0	7	7
Proteobacteria	*Bradyrhizobium*	30	0	13	29	0	nd	13
Fusobacteria	*Sneathia*	31	0	7	25	0	7	0

* Percentage of samples that contained the genus.

† Disagreement between paired EST and biopsy samples.

In bold are the genera significantly different between the biopsy and the EST.

This table lists the top 20 genera in rank of median relative abundance. Also featured are the percentage detection and disagreement from the 15 individual biopsies and ESTs, (nd, not detected).

### Bacterial communities are different in the oral, nasal, and esophageal microenvironments

**Figure 6:**
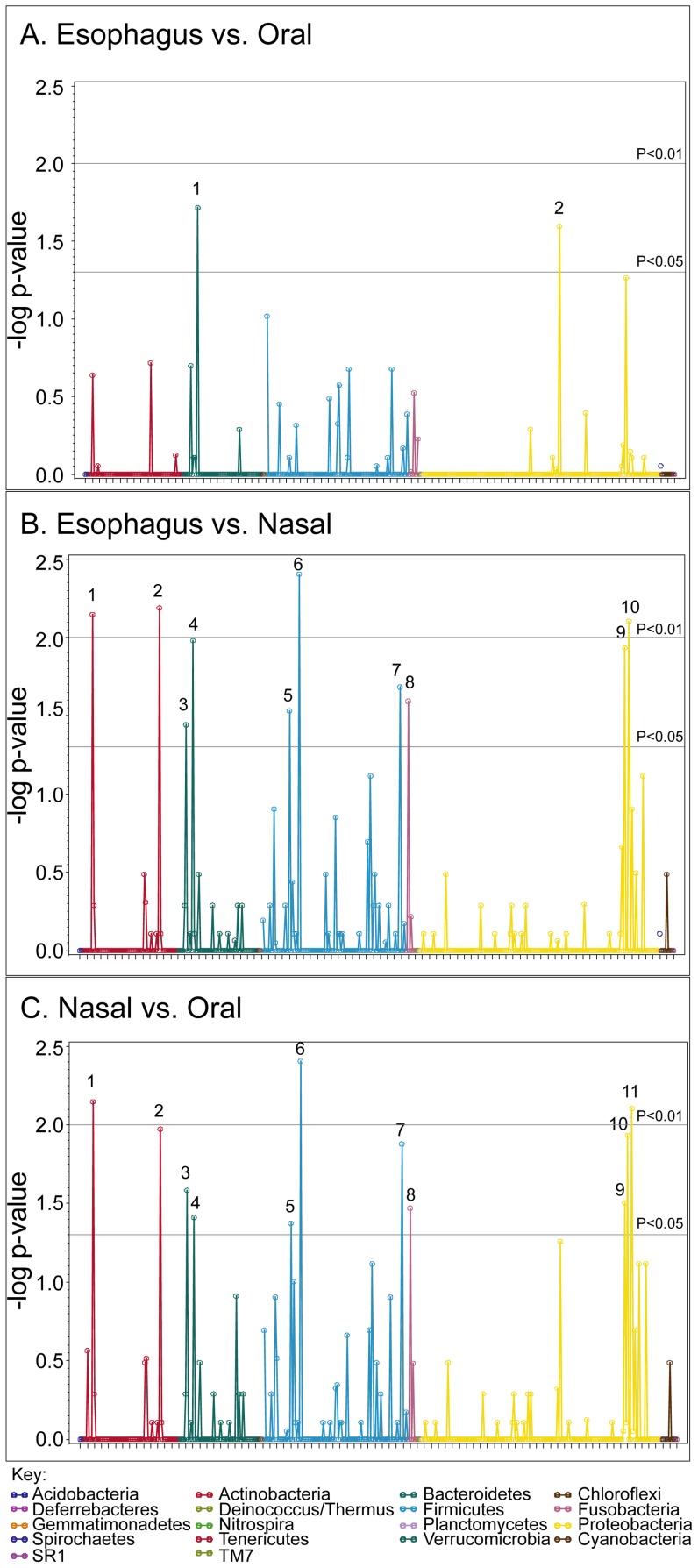
Oral and nasal microbiomes differ from the esophageal microbiome. DNA samples were taken from the oral, nasal and EST and the microbiome identified using 454 pyrosequencing. Bacterial phyla are compared between biopsy and EST samples and results displayed using Manhattan plot as follows. A. Esophagus (EST) compared to the oral cavity, Genera that were significantly different are numbered. Numbers represent: 1, *Prevotella*; 2, *Neisseria*. 1, had higher relative abundance in EST. B. Esophagus (EST) compared to the nasal cavity. Numbers represent 1, *Corynebacterium*; 2, *Propionibacterium*; 3 *Porphyromonas*; 4, *Prevotella*; 5, *Staphylococcus*; 6, *Streptococcus*; 7, *Veillonella*; 8, *Fusobacterium*; 9, *Haemophilus*; 10, *Pasteurella*. Numbers 3, 4, 6–10 were higher in EST. C. Nasal cavity compared to the oral cavity. Numbers represent: 1, *Corynebacterium*; 2, *Propionibacterium*; 3, *Porphyromonas*; 4, *Prevotella*; 5, *Staphylococcus*; 6, *Streptococcus*; 7, *Veillonella*; 8, *Fusobacterium*; 9, *Aggregatibacterium*; 10, *Haemophilus*; 11, *Pasteurella*. Numbers 3, 4, 6–11 were higher in oral samples. Gray horizontal lines indicate significant differences (p<0.05 and p<0.01). Phyla are represented on the X-axis as different colors as in the key.

We defined the bacterial community of each microenvironment captured by the string and swabs. Comparisons of the microbiome captured in oral cavity and EST samples reveal two genera differences (Figure 6A); *Prevotella* was found in significantly higher amounts in the EST (P = 0.002), whereas *Neisseria* was significantly lower in ESTs (P = 0.03). In addition, comparisons of the microbiome captured in the nasal cavity and EST samples revealed significant differences across several phyla and genera (Figure 6B). Finally, results with the nasal and oral microbiome revealed multiple differences in these two locations (Figure 6C). These results suggest that each of these microenvironments harbor specific taxa that distinguish the sites, and importantly, that the esophageal segment of the EST does not become contaminated by the oral microbiome upon removal.

## Discussion

As the role of the microbiota in the pathogenesis of disease becomes increasingly evident, there is need for development of new and less invasive tools with which to sample microbiomes in specific microenvironments throughout the body [1]. Use of mucosal biopsies has contributed to an emerging body of data identifying changes in esophageal microbiome in both health and disease [4], [5], [6], [7], [8], [22]. In this study, we surveyed the microbiome associated with normal non-inflamed mucosal biopsy samples the “gold standard” and the proximal section of the Enterotest or Esophageal String Test (EST). Results from our studies contribute to a growing body of research defining the esophageal microbiome and provide strong evidence that the microbial composition captured by the EST accurately reflects the microbiome as compared to mucosal biopsy samples. The EST technique is less costly and incurs less risk, while potentially providing a more comprehensive view of the esophagus than is obtained with a more limited, single ∼3 mm mucosal biopsy sample, as the EST interrogates a significantly greater surface area of the esophagus. We conclude that the EST is an appropriate tool with which to assess the esophageal microbial microenvironment.

Several methods have been used to capture esophageal microbiome samples but all have utilized endoscopy. Previous studies measuring the esophageal microbiome from mucosal biopsy sampling identified patterns of remarkable similarity to that identified with the EST [4]. Consistent with these studies, our results reveal that the predominant genera in the esophagus are *Streptococcus*, *Prevotella* and *Veillonella* [3], [4], [5], [6], [8]. Esophageal aspirates and esophageal brushing were able to capture similar bacteria [6] [5]. While these later two techniques seek to procure mucosal samples without biopsy, they both require upper endoscopy to obtain samples. Thus, while each of these techniques captures similar bacteria to the EST, the EST offers the benefit of its minimally invasive nature.

Traditional culture techniques have been used to identify the makeup of the esophageal microbiome, thus, leaving non-cultivable bacteria unidentified [23]. Our study took advantage of culture-independent sequencing techniques that are capable of identifying many organisms in parallel, without prior knowledge of growth requirements. This technique provides a more complete catalog of the esophageal microbial community as a reference for future studies.

Direct comparisons of the microbiome in the nasal, oral and esophageal cavities have not been previously reported. While one might expect that the intimate anatomical connections of these organs would lend to their possessing similar microbiomes, our results and that of others have indicated that the nasal microbiome [24], [25] is distinct from the oral and esophageal microbiomes [5]. Our study compared all three bacterial assemblages simultaneously, and found that despite some similarities between sites, distinct differences occur in the normal, oral and nasal cavity, and the esophagus. Whether this trend holds during inflammatory processes, awaits further study.

Over the last decade, a growing body of knowledge has demonstrated the influence of the gastrointestinal microbiome on health and disease. For instance, alterations of the colonic microbiome have been associated with Crohn's disease as well as obesity [26], [27], [28], [29]. Although such studies indicate potential roles of the colonic microbiome in colonic disease, much less is known about the role of the microbiome in the esophagus. A critical issue has been access to specimens for analysis of esophageal microbiology. In contrast to the relative ease in obtaining fecal samples, human studies of the rest of the gastrointestinal tract rely primarily on samples obtained through mucosal biopsies at the time of endoscopy. Repeated sampling of anatomical spaces is required to address stability since the microbiome can change over time and with treatments [30], [31]. Access to esophageal luminal contents with minimally invasive collection, as with the EST, will allow more sampling opportunities for exploring upper gastrointestinal tract diseases.

In the current study, some of the subjects were on various treatments (PPI, steroids, restricted diet) at the time of sample collection that could affect the microbiome as compared to normal individuals without any treatment: e.g., the use of PPIs was found to increase the bacterial load on esophageal biopsies [32]. Furthermore, steroid responsiveness has been associated with the microbiome in inflammatory bowel diseases [33]. Diet is another factor that is known to influence the microbiota [28], [34]. Dentogingival health may also affect the oral microbiome [35] and potentially the esophageal microbiome. However, the goal of this study was to determine if the EST could accurately reflect the mucosal microbiome, and we did not have adequate power to determine the effect of a specific treatment on the esophageal microbiota. Larger studies, facilitated by the EST, will address this issue as adequate numbers of subjects without esophageal involvement are recruited.

Overall, the EST was well tolerated in our study, with gagging noted as the only side effect in some subjects. Contamination from adjacent sites is possible, but our results suggest clear differences in the microbiota. Consistent with previous studies our results show that, some bacteria found in the normal oral cavity, such as *Spirochetes* and *Deferribacteres* [36], are not conspicuous in the normal esophagus [5]. Our study included a larger number of females than males, but we saw no influence of gender on our findings. However, it has been reported that in the absence of disease, similar phylum level patterns are observed in the esophageal and gastric compartment independent of ethnicity or gender [36].

Rare genera were detected more frequently in biopsy samples than on the EST, and there are several possible explanations for this observation. The EST is able to capture microbiome from a greater esophageal surface area, and thereby increase the signal from common genera and making detection of rare genera less frequent with EST samples. Further, it is possible that different taxa have variable affinities for the mucosa, perhaps affecting the ability of the string to capture them. Additionally, the overnight placement of the string in the esophagus may allow particular genera to proliferate and thereby alter relative abundances seen molecularly.

In conclusion, the EST is a novel, minimally invasive device with which to sample the esophageal microbiome, with results comparable to those obtained with esophageal biopsies. Use of the device is particularly appropriate for children, as it obviates the need for endoscopy and anesthesia. Easier sampling of the esophageal lumen using the EST will facilitate future studies of the esophageal microbiome in disease and health.

## Supporting Information

Figure S1 **Esophageal String Test.** The Enterotest™ capsule with the extruded string is shown. The top arrow shows the portion of the string that is taped to the cheek. The bottom arrow indicates the weighted capsule that is dislodged in the duodenum.
(TIF)

Figure S2 **16S amplification and detection of oral, nasal and esophageal microenvironments.** This is a picture of a representative 2% agarose gel with a 200 bp amplification product from the V2–V3 region of the 16S rDNA gene. DNA samples were obtained from nasal swabs, oral strings, ESTs and esophageal biopsies.
(TIF)
