# Supplementary figures and images for: Novel Device to Sample the Esophageal Microbiome—The Esophageal String Test
Source: PLoS One. 2012 Sep 5;7(9):e42938. doi: 10.1371/journal.pone.0042938 (PMC3434161; doi:10.1371/journal.pone.0042938)

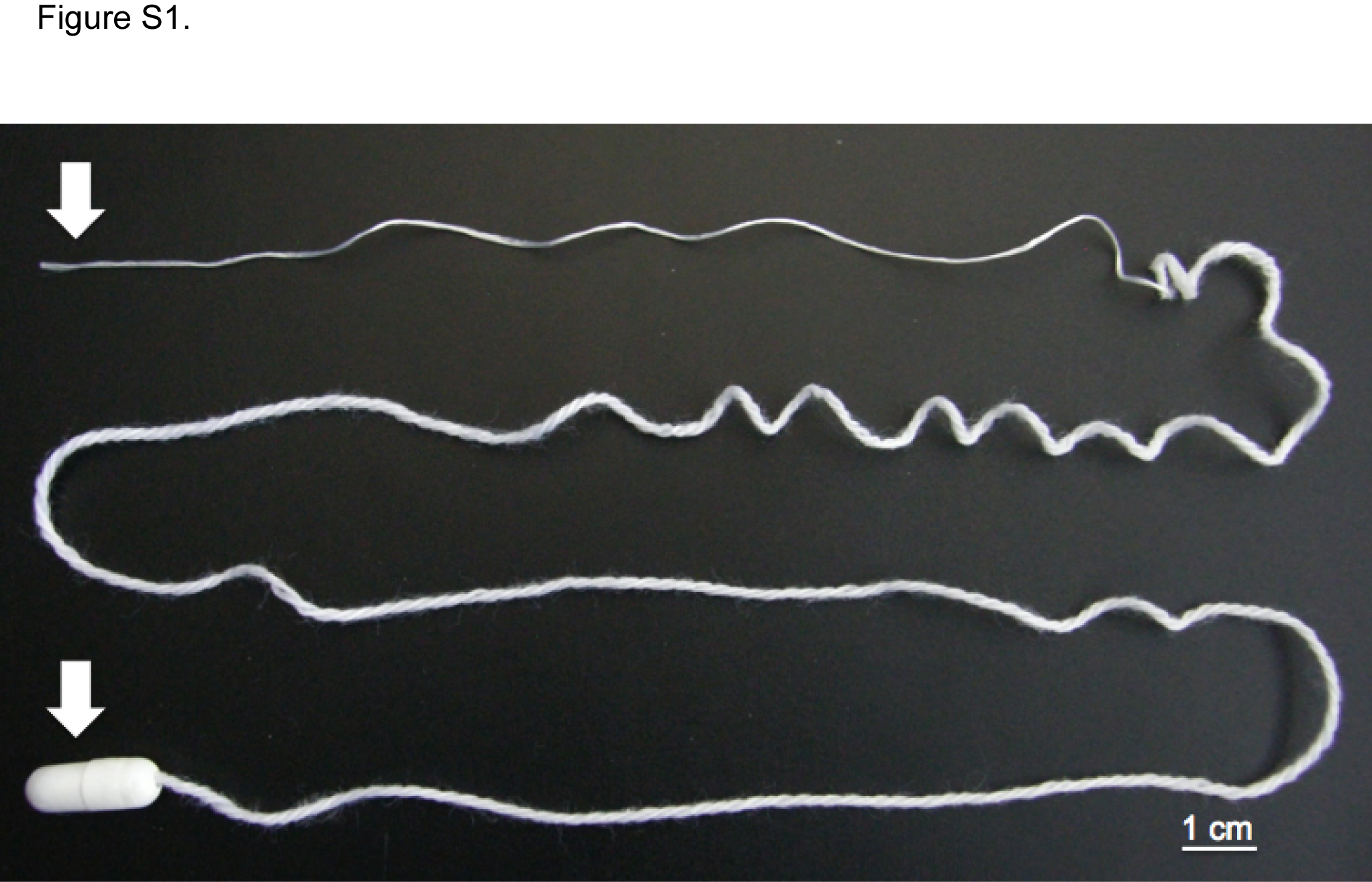

Supplement: Figure S1 — Esophageal String Test. The Enterotest™ capsule with the extruded string is shown. The top arrow shows the portion of the string that is taped to the cheek. The bottom arrow indicates the weighted capsule that is dislodged in the duodenum. (TIF) [file pone.0042938.s001.tif]

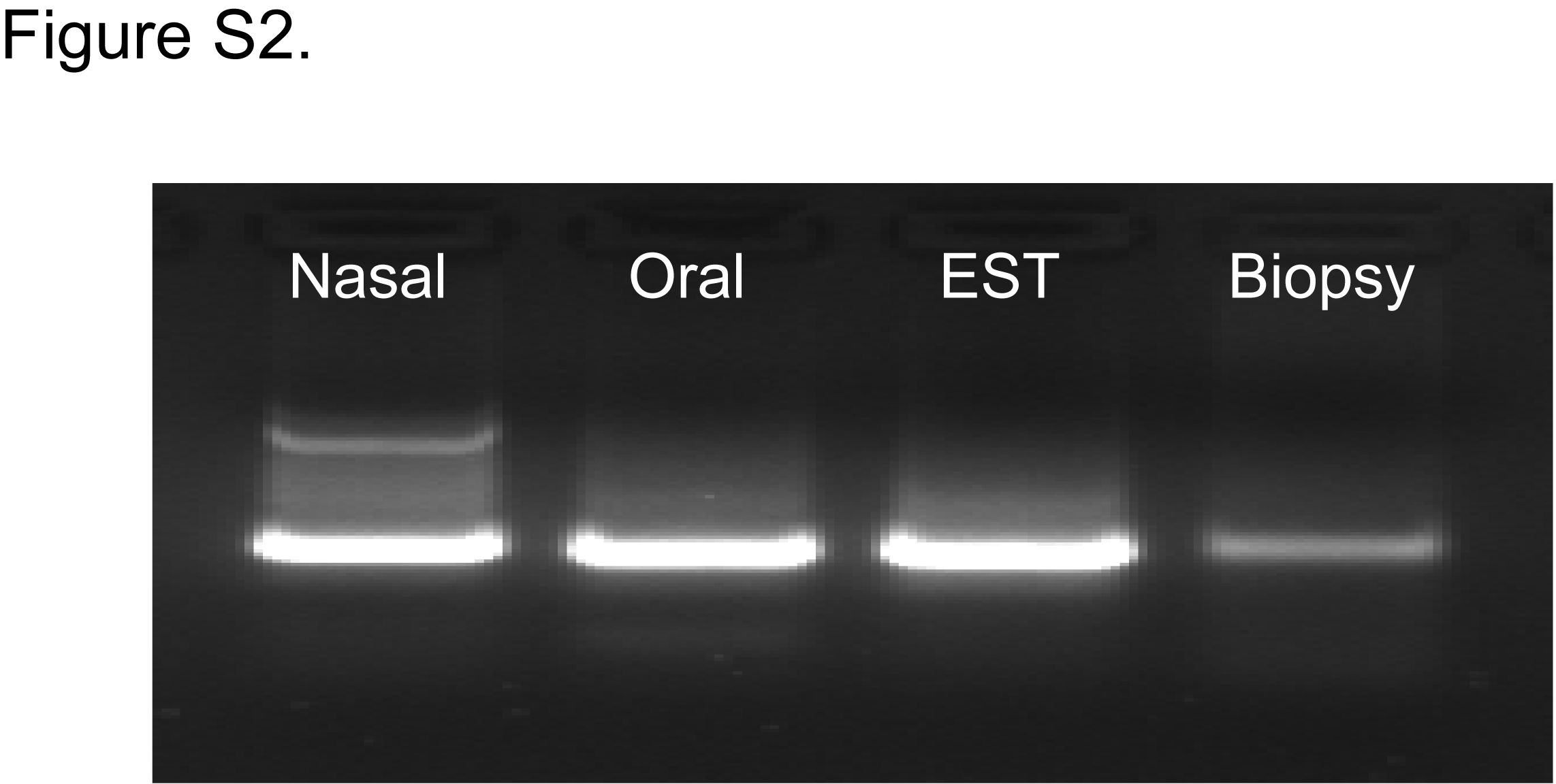

Supplement: Figure S2 — 16S amplification and detection of oral, nasal and esophageal microenvironments. This is a picture of a representative 2% agarose gel with a 200 bp amplification product from the V2–V3 region of the 16S rDNA gene. DNA samples were obtained from nasal swabs, oral strings, ESTs and esophageal biopsies. (TIF) [file pone.0042938.s002.tif]
